# Trauma in the lives of parents experiencing severe perinatal mental illness

**DOI:** 10.3389/fpsyt.2024.1380146

**Published:** 2024-04-02

**Authors:** Sophie Isobel

**Affiliations:** ^1^ Naamuru Parent and Baby Unit, Sydney Local Health District, Sydney, NSW, Australia; ^2^ University of Sydney, Faculty of Medicine and Health, Sydney, NSW, Australia

**Keywords:** complex trauma, trauma informed care, perinatal mental illness, mother baby units, mentalisation, attachment

## Abstract

**Background:**

The perinatal period is a time of ‘high risk’ for new and recurrent episodes of mental illness with 0.1-0.2% of birthing parents requiring admission to specialist mental health units in the months after birth. The prevalence and role of trauma in the lives of birthing parents (most commonly mothers) experiencing severe perinatal mental illness is not well known.

**Method:**

In a new perinatal mental health unit in Sydney Australia, a retrospective audit of trauma prevalence was undertaken using patient completed questionnaires and electronic medical record data. Descriptive analysis was undertaken.

**Results:**

Prevalence of trauma in the lives of mothers with severe mental illness was found to be higher than that reported in general or community mental health settings, with 76% of mothers reporting lifetime trauma exposure and 24% meeting criteria for complex PTSD. The majority reported trauma experiences likely to impact attachment and also reported difficulties in responding to their infants’ cues and needs.

**Discussion:**

The findings suggest a need for more research, awareness, and consideration of the role of trauma in experiences of perinatal mental illness, with implications for developing trauma informed models for responding to parental mental illness.

## Introduction

The perinatal period is a period of tumult and change for many parents. The postnatal period in particular is considered a time of ‘high risk’ for new and recurrent episodes of mental illness, with around one to two birthing parents in every thousand requiring admission to a mental health unit in the months after birth (1). Contributing factors to perinatal mental illness include physiological, existential, relationship, financial and social changes. Many parents also report demoralization, feelings of helplessness and a sense of incompetence during early parenthood (2). Mental illness in the perinatal period is correlated to maternal and infant morbidity and mortality (3, 4), lifetime disadvantage for families and childhood adversity (1, 5), with links also often made to intergenerational distress and illness (6, 7). There is also suggested high prevalence of experiences of developmental or lifetime trauma in parents experiencing perinatal mental illness (8, 9).

Trauma does not refer to one thing. Trauma is encompassing of a wide variety of events, experiences and effects, usually involving fear or betrayal, that can impact in differing ways (10). Complex trauma refers to trauma that is cumulative, usually developmental and interwoven into attachment relationships. Experiences which lead to complex trauma often involve strong feelings of fear and powerlessness within stressful or chaotic environments, where flexible coping strategies aren’t effective (10). Examples include experiences of childhood abuse or neglect by caregivers. While effects of trauma are often assumed to be directly linked to the event (for example, fear of dogs after a dog attack), in complex trauma, the effects are more ‘complex’ because they are woven into perceptions of self, others and the world. Complex trauma is distinguishable by lifelong disturbances in self-organization, most prominently in responding to, displaying, and regulating strong emotions (10).

Trauma is not always directly correlated to perinatal distress (11, 12), yet, knowledge of the ways developmental trauma can affect attachment, self-regulation, health and relationships (10) suggests that it is plausible that experiences of perinatal mental illness may be more complicated by trauma, particularly attachment or relational trauma. Attachment theory posits that early relational experiences shape the attachment system and influence patterns of attachment across the lifespan. Complex trauma in particular is likely to be highly relevant to the perinatal period due to activation of the attachment system (13, 14). During the transition to parenthood a reorganization of self occurs, a period during which the attachment system is constantly activated (15). Complex trauma is known to impact upon mentalisation, infant regulation and parental self-concept (16); however, the role of complex trauma in the lives and recovery of parents experiencing perinatal mental illness remains not well understood or addressed in services. Mentalisation refers to the capacity to think about, understand, interpret, and predict one’s own and others’ behaviour, with reflection on underlying mental states and intentions, such as feelings, thoughts and intentions (17). Mentalisation involves reflective capacity, whereby parents maintain a psychological model of the infant as a separate psychological being with a mind and mental states, reflected through sensitive and attuned interactions and supporting the infant to remain within an optimal range of physiological arousal (12).

Mother Baby Units (MBUs) provide specialist inpatient mental health care for a small percentage of parents experiencing moderate to severe mental illness in the perinatal period. MBUs are mental health or psychiatric units. The need for MBUs has been well established; MBUs are considered ‘best practice’ for improving outcomes for parents and babies when the primary caregiver (usually the mother) is experiencing severe perinatal mental illness (3). Across studies, parents admitted to MBUs have demonstrated improved mental health, relationships with their infants and parenting confidence on discharge through mental health care and concurrent parent-infant interventions (1, 8, 18, 19). All parent-infant interventions are embedded in understandings of attachment theory and the neuroscience of early infant brain development, as well as the significance of early stress and trauma on infant development (20). However, there has been remarkably little research on the relationship of trauma to perinatal mental illness and how MBUs can best support mothers with complex mental health needs, interwoven with trauma and attachment. There is a need to understand the prevalence and intergenerational impacts of trauma associated with severe perinatal mental disorders (12, 21, 22), specifically in the context of inpatient perinatal mental health care.

## Materials and methods

This study uses existing clinical data to explore the prevalence and possible impacts of trauma upon mothers experiencing severe perinatal mental illness in Sydney, Australia, for the purposes of a considering the role of service providers in providing attachment and trauma-informed mental health care.

A new MBU was established in inner Sydney, Australia, in 2022. For the first year of operation, the unit provided the only public inpatient perinatal mental health care in the state. The unit is an 8-bed acute mental health unit, delivering 24-hour mental health care to parents, alongside support for infants and a support person. Following the first 12months of operation, retrospective clinical data from the cohort of parents admitted to the unit were descriptively analysed to explore demographics, rates of reported potentially traumatic experiences in parents and to explore the relationship between types of traumatic events and perceived responsiveness to infant cues. No inferential statistics or tests of significance were undertaken in acknowledgment of the complexity and overlap of the key concepts of trauma and attachment, and to avoid hierarchical or linear interpretation. The study had ethics approval from the local hospital ethics committee (X22-0198 2022_ETH01237).

Parents are referred from mental health services across the state to the MBU, when community-based care is not sufficient, and an acute mental health admission is warranted. In the first 12 months of the service, from June 2022-June 2023, 74 parents (all identifying as women) were admitted to the unit, aligning to expected rates of severe perinatal mental illness in the population. The majority were admitted with a referring diagnosis of perinatal depression, anxiety or psychosis. Retrospective trauma screening was performed for all admitted patients (N74) through data points retrieved from subjective patient completed questionnaires and objective extraction from the electronic medical record.

Patients completed the questionnaires on an electronic device as part of admission to the unit. Patients were informed as part of the questionnaires that de-identified data would be used for evaluation and research; however, a waiver of consent was granted for all clinical data used. Patient questionnaires included the Post-Natal Risks and Vulnerability Questionnaire (PNRQ; (23) and the Karitane Parenting Confidence Scale (KPCS; (24). Data from the medical record were extracted from referral, admission notes, progress notes and discharge summaries, with any report of trauma or developmental trauma noted. Data points linked to trauma were identified from across the questionnaires and medical record, binarized to ‘identified trauma’ or ‘not identified trauma’ and calculated for percentages of the study population, with some parents reporting more than one form of abuse. When ambiguity was present, ‘no identified trauma’ was selected. Types of abuse were extracted from the self-report PNRQ which asks about sexual, physical, emotional abuse and lack of maternal support during development. Mentalisation was proxied through extracted questions from the self-report KPCS, analysed across traumatic events. Forty-six parents completed a PNRQ and KPCS on admission (62% of cohort). A primary or secondary diagnosis of complex trauma was identified from discharge summaries completed by the unit psychiatrists.

All data were deidentified for analysis using a clinical database in REDCAP. Percentages and total numbers were manually calculated for each variable.

## Results

Prevalence of trauma in the cohort was calculated to be 76%, with 66% reporting developmental trauma from their own childhood, including high rates of sexual abuse and lack of maternal support. Almost a quarter of the cohort met diagnostic criteria for complex PTSD upon discharge. See **Table 1**.

**Table 1:** trauma prevalence.

Self-report trauma screening (PNRQ)	% (n)
Emotional abuse during development	30% (22)
Physical abuse	31% (23)
Sexual abuse	47% (35)
Lack of maternal emotional support during development	46% (34)
Trauma screening from medical records	
Trauma identified	76% (56)
Developmental/attachment trauma	66% (49)
Complex trauma diagnosis on discharge (primary or secondary)	24% (18)

When observing the responses of women who identified an overt type of abuse in their lifetime, in contrast to women with no reported abuse (See **Table 2**), women who had experienced abuse seemingly reported more difficulty in soothing their babies and more difficult in understanding the baby’s cues despite comparable or higher rates of reported affection towards the baby.

Table 2Responsiveness to infant’s cues.

**Table:** 

I can soothe my baby when they are distressed: %(n) N46				
	Emotional abuse n(19)	Physical abuse n(15)	Sexual abuse n(21)	No reported abuse n(15)
Not at all or not often	21%(4)	20% (3)	14.3% (3)	6.7% (1)
Some or most of the time	79% (15)	80% (12)	85.7% (18)	93.3% (14)

**Table:** 

I understand what my baby is trying to tell me: %(n) N46				
	Emotional abuse n(19)	Physical abuse n(15)	Sexual abuse n(21)	No reported abuse n(15)
Not at all/Not very often	36.9% (7)	33.3% (5)	33.3% (7)	6.7% (1)
Some or most of the time	63.1% (8)	66.7% (10)	66.7% (14)	93.3% (14)

**Table:** 

Over the last 2 weeks, I would describe my feelings for the baby as: %(n) N46				
	Emotional abuse n(19)	Physical abuse n(15)	Sexual abuse n(21)	No reported abuse n(15)
Dislike	0%(0)	0% (0)	0% (0)	0% (0)
No real affection	15.8% (3)	6.7%(1)	14.3% (3)	6.7%(1)
Slight affection	10.5%(2)	20%(3)	14.3% (3)	13.3%(2)
Moderate affection	26.3% (5)	26.7% (4)	23.8% (5)	46.7%(7)
Intense affection	47.4% (9)	36.8% (7)	47.6% (10)	33.4%(5)

## Discussion

As the only MBU in the state during the study period, this small single site study explored the prevalence of trauma in the population of women requiring inpatient mental health care for perinatal mental illness over a 12-month period in mid-2022 to mid-2023. The results suggest high rates of exposure to traumatic events amongst this patient group admitted to an inpatient unit with severe mental illness in the perinatal period, with the majority having developmental trauma experiences likely impacting attachment processes.

Identified prevalence rates of trauma in this group are much higher than rates in the general community, where for example, studies have found lifetime prevalence of interpersonal trauma or childhood trauma between 11.8% (25) and 27.7% (11) in pregnant women in general antenatal settings. Women experiencing perinatal mental illness and receiving community-based care have been found to have rates of childhood trauma closer to the current study, between 34% (26) and 60% (27). This finding suggests a possible relationship between trauma prevalence and severity of perinatal mental illness, and a need for greater understanding of this relationship. Exposure to any of the potentially traumatic past events screened for in this study cannot be assumed to result in ongoing traumatic effects in the present, as how people respond to trauma is inherently complex and multifactorial. Yet, almost a quarter of women in the current patient group had a diagnosis of complex trauma upon discharge, indicative of functional impacts, despite community prevalence of complex trauma estimated to be 0.5% (28). Women in the current study who had experienced abuse appeared more likely to have difficulty in interpreting their babies’ needs and soothing babies’ distress, despite feelings of affection. It is possible that this reflects a disruption to mentalisation. Women with a history of trauma are known to be more likely to experience both adult mental illness and attachment disturbances with their infants, including activation of traumatic memories or emotions in the context of caregiving leading to decreased sensitivity and responsiveness (12, 29, 30) and overall poorer quality interactions (31). Emotional regulation is thought to play a key role in this link between trauma and mental illness (32, 33), through altered development of affect regulation and stress modulation and recognised intergenerational patterns (34). While direct associations between trauma, perinatal mental illness and intergenerational attachment are always difficulty to identify, past and current experiences of trauma are more strongly linked to attachment difficulties than perinatal mental illness (35); with parents with childhood abuse or adult domestic violence and perinatal mental illness presenting as more withdrawn, less playful, and less responsive than other parents also experiencing perinatal mental illness (35). Trauma itself does not compromise parental sensitivity. Rather, a parent’s capacity to consider how their own experiences of trauma may impact their ability to be sensitive and responsive to their infant (36). Mentalisation is thus thought to be the most effective interventional target for improving attachment quality (22), with prevention of intergenerational trauma transmission requiring resolution of trauma and concurrent attachment interventions, targeting mentalisation (22). The findings of the current study suggest a need for mentalisation-focused interventions alongside mental health treatment during MBU admissions for mothers with severe mental illness, particularly those who report experiences of trauma. Any attempt to ‘measure’ trauma, particularly complex types, is complicated. It relies on cohesive narratives of events which contradicts what is known about the dynamics of traumatic defences, or upon assessment of symptoms using diagnostic categories which only identify people who display specific pathways of response (10). It also relies on shared understandings of what trauma is. In this study, potentially traumatic events were considered through an attachment lens, with a focus on experiences of abuse, emotional neglect or childhood adversity and their relevance to feelings and responses to infants. While awareness and sensitivity to other forms of trauma is required across health care settings, in perinatal settings, clear definition of developmental, attachment and/or complex trauma is essential to identification and formulation.

Increasingly all mental health services are recognising a need to become ‘trauma-informed’ due to the prevalence of trauma in the lives of people experiencing mental distress, as well as the implications for treatment and recovery (37). Trauma-informed approaches to care require clinicians and services to consider that all individuals accessing their service may have experiences of trauma, that having a mental illness can be traumatic, that there is risk of psychological harm occurring in care, and that there are ways that care can be structured and delivered to be sensitive to trauma (38). However, efforts towards trauma-informed approaches have been hampered by disagreement about the relationship between trauma and mental illness and dominance of biological models of psychiatry (39). While trauma-informed approaches are universally indicated, in perinatal mental health settings trauma-informed approaches need to also overtly address trauma to support parents to consider how their own experiences of past trauma may impact their ability to be sensitive and responsive to their infants in the present.

The very high rates of trauma in this MBU patient group suggests that while the literature reflects evidence of the benefits of MBUs (18, 19), there remains a need to understand their potential role beyond treating parental mental illness within existing psychiatric models. MBUs are one setting where resolving tensions between biological and trauma-informed approaches is urgently required. The current study findings reinforce calls by Howard and Khalifeh for developing and evaluating multi-generational, trauma-informed perinatal mental health care models (1). Supporting parents to think about and symbolise traumatic events and to consider their impacts in psychological terms may reduce their own reactivity to their infant and enable them to remain able to soothe their infant despite the infant’s distress triggering their own traumatic memories (22). While this requires longer-term engagement, proactive support with establishing and maintaining attachment relationships in MBUs is essential to ensuring that the relational space can adequately buffer and protect infants from the intersubjective transmission of trauma sequelae (22). The perinatal period is a time for the establishment of neurological and psychological capacities and functioning, with direct relationships upon lifetime mental health (20) and as such, translating awareness of trauma and its impacts upon attachment to the delivery of perinatal mental health care, including in MBUs, is essential.

### Limitations

As a retrospective study using existing clinical data, the current findings can only be interpreted to suggest a need for much more consideration of the role of trauma (particularly those forms which are commonly interwoven with attachment) in experiences of perinatal mental illness, both in the context of prevention of distress and when considering intergenerational impacts through attachment and mentalisation. No inferential statistics were used in this study because the focus was on exploring prevalence and proposing possible clinical significance, rather than determining (or distracting with) statistical probability or significance (40) which is recognised to be of limited ‘real world’ utility (41). The sample size was small, and the design further limited by the ‘messiness’ of existing health data. Examining existing data can reduce burden on participants in healthcare research and guide the focus of future studies, but it can also be incomplete, inaccurate and lacking sensitivity. There are several trauma and mentalisation specific measures which were not included in the patient questionnaires used for clinical purposes on the MBU. Future research would benefit from the use of effect size calculations, validated complex trauma and mentalisation tools such as the International Trauma Questionnaire (42) and the Reflective Functioning Questionnaire (43), as well as qualitative exploration of mothers’ perspectives.

### Implications

There have been calls for a paradigmatic shift in perinatal mental health from focusing on treating maternal mental illness with the infant present, to centring the conflicts of transitioning to parenting, the interactional aspects of care and the role of transgenerational attachment (20). MBUs are one key site where this is applicable, but it also impacts any setting where parents access mental health care. Despite significant efforts towards trauma-informed approaches in mental health settings broadly, responses to trauma are known to be inconsistent and largely avoided (39, 44, 45).

Relational traumas can be transmitted across generations through parent–infant relationships, with intergenerational trauma considered both an antecedent and outcome of traumatic attachment (22, 34, 46). Yet, similar to mental illness more broadly, not all parents with trauma will pass on traumatic effects to the next generation, and not all trauma-related intergenerational effects will be problematic (47). Subsequently, a trauma-informed lens should not focus purely on screening and identification but on how parents can be supported to recognise and adapt to the impacts of traumatic experiences and effects upon their parenting, attachment and perinatal mental health.

## Data availability statement

The raw data supporting the conclusions of this article will be made available by the authors, without undue reservation.

## Ethics statement

The studies involving humans were approved by Royal Prince Alfred Hospital Human Research Ethics Committee. The studies were conducted in accordance with the local legislation and institutional requirements. The ethics committee/institutional review board waived the requirement of written informed consent for participation from the participants or the participants’ legal guardians/next of kin because Retrospective medical record audit only, no direct recruitment of participants to minimise coercion and burden of people experiencing mental distress.

## Author contributions

SI: Writing – original draft, Writing – review & editing.
