# Erratum: Trauma in the lives of parents experiencing severe perinatal mental illness

**DOI:** 10.3389/fpsyt.2024.1453853

**Published:** 2024-07-03

**Authors:** Frontiers Production Office

**Affiliations:** Frontiers Media SA, Lausanne, Switzerland

**Keywords:** complex trauma, trauma informed care, perinatal mental illness, mother baby units, mentalisation, attachment

Due to a publisher error, this article was incorrectly published as an Original Research article when it should have been a Brief Research Report article.

The publisher apologizes for this error. The original article has been updated.

